# A Phase-II Investigator-Initiated Randomized, Double-Blind, Parallel-Group Clinical Trial of 5-Aminolevulinic Acid Hydrochloride/Sodium Ferrous Citrate (5-ALA-HCl/SFC) for Patients With Adult-Onset Still’s Disease (AOSD) Refractory to Corticosteroids

**DOI:** 10.7759/cureus.90354

**Published:** 2025-08-17

**Authors:** Remi Sumiyoshi, Tomohiro Koga, Osamu Kamisawa, Shimpei Morimoto, Naoki Hosogaya, Hiroshi Yamamoto, Mariko Kawakami, Yosuke Inaba, Yoko Hattori, Tamiki Hikake, Tomoya Kurokawa, Hideki Hanaoka, Shuzo Sato, Kiyoshi Migita, Noriyoshi Ogawa, Motomu Hashimoto, Atsushi Kawakami

**Affiliations:** 1 Immunology and Rheumatology, Division of Advanced Preventive Medical Sciences, Nagasaki University Graduate School of Biomedical Sciences, Nagasaki, JPN; 2 Clinical Research Center, Nagasaki University Hospital, Nagasaki, JPN; 3 Clinical Research Center, Chiba University Hospital, Chiba, JPN; 4 Department of Rheumatology, Fukushima Medical University School of Medicine, Fukushima, JPN; 5 Department of Rheumatology, St Francisco Hospital, Nagasaki, JPN; 6 Internal Medicine 3, Division of Immunology and Rheumatology, Hamamatsu University School of Medicine, Hamamatsu, JPN; 7 Clinical Immunology, Osaka Metropolitan University of Medicine, Osaka, JPN

**Keywords:** 5-aminolevulinic acid hydrochloride/sodium ferrous citrate (5-ala-hcl/sfc), adult-onset still's disease, glucocorticoids, heme oxygenase (ho-1), systemic feature score

## Abstract

Objective: To evaluate the efficacy and safety of 5-aminolevulinic acid hydrochloride/sodium ferrous citrate (5-ALA-HCl/SFC) in patients with adult-onset Still’s disease (AOSD) refractory to corticosteroids.

Methods/design: This multicenter, investigator-initiated, randomized, double-blind, placebo-controlled, parallel-group phase II trial was designed to enroll 30 participants but was prematurely terminated after enrolling four participants because of slow recruitment. Participants were randomized to receive 5-ALA-HCl/SFC (100 or 300 mg/day) or placebo for 8 weeks. The primary endpoint was the achievement of adapted ACR 30 at week 4.

Results: All four enrolled participants achieved adapted ACR 30 at week 4. Adapted ACR 50/ 70/ 90 responses and improvements in systemic feature score, serum ferritin, and quality of life varied among individuals. No serious adverse events were observed.

Conclusion: Efficacy signals were observed, especially in the high-dose group; however, the small sample size precludes definitive conclusions. Further research is required to confirm this.

## Introduction

Adult-onset Still’s disease (AOSD) is a systemic inflammatory disorder of unknown cause that develops at age 16 years or older and accounts for 95% of adult Still’s disease [1]. The clinical features are varied and nonspecific, with fever exceeding 39°C, asymmetric arthralgia/arthritis, skin rash (salmon pink rash), sore throat, enlarged lymph nodes, hepatosplenomegaly, and inflammatory findings (neutrophilic leukocytosis, increased erythrocyte sedimentation rate (ESR), elevated C-reactive protein (CRP), impaired liver function, and hyperferritinemia) [1,2]. Although the recent European Alliance of Associations for Rheumatology/Paediatric Rheumatology European Society (EULAR/PReS) guidelines recommend biologics as the first-line treatment [3], in Japan, initial therapy frequently starts with corticosteroids. While the overall prognosis is generally favorable, about 60% of patients experience a protracted disease course. Therefore, long-term issues include poor quality of life and activities of daily living associated with joint destruction, metabolic complications (diabetes, arterial stiffness, osteoporosis, etc.) associated with long-term corticosteroid medication, and secondary amyloidosis associated with persistent inflammation.

The 5-aminolevulinic acid (5-ALA) is an amino acid and a precursor of porphyrin metabolism in plants and animals. In cells, protoporphyrin IX (PpIX) is synthesized from eight molecules of 5-ALA through several reactions, and ferrochelatase inserts iron (II) into PpIX to produce heme. The simultaneous administration of 5-ALA-HCl and sodium ferrous citrate (SFC) (5-ALA-HCl/SFC) promotes the conversion of PpIX to heme [4]. Preclinical studies using Balb/c mice and Sprague-Dawley rats have shown that oral administration of 5-ALA-HCl/SFC induces the expression of heme oxygenase-1 (HO-1) by increasing intracellular heme levels. HO-1 is predominantly expressed by macrophages and endothelial cells in response to various stresses [5]. It is also called heat shock protein-32, is a 32kD heme-degrading enzyme, and has been reported to have anti-inflammatory effects [6,7]. It is effective in ischemia-reperfusion injury, cisplatin-induced nephropathy, and other inflammatory diseases [8,9]. It has been shown that 5-ALA-HCl alone does not induce sufficient expression of HO-1 and has insufficient therapeutic effect on the above-mentioned inflammatory diseases, and simultaneous administration of 5-ALA-HCl and SFC is considered necessary to exert an anti-inflammatory effect [8-10].

 Elevated levels of HO-1 have been reported in the sera of patients with AOSD. Its dynamics parallel those of serum ferritin, which is conventionally used as a marker of disease activity in AOSD. There is a positive correlation between serum ferritin levels and HO-1 levels in patients with AOSD. The fact that HO-1 expression decreases with treatment suggests that it contributes to the pathogenesis of the disease [11]. In AOSD, HO-1 is thought to be induced in response to stress, such as cytokine storms, functioning as a self-limiting mechanism. Therefore, the increase in HO-1 is considered to be a consequence of severe inflammation rather than a primary cause.

Based on these findings, we hypothesized that 5-ALA-HCl/SFC could be a potential therapeutic agent for AOSD. Therefore, we conducted this phase II study to confirm the efficacy of 5-ALA-HCl/SFC in patients with AOSD.

## Materials and methods

Study design

This was an investigator-initiated, multicenter, phase II, double-blind, randomized, parallel-group comparison study of the efficacy and safety of 5-ALA-HCl/SFC compared with placebo in patients with AOSD refractory to corticosteroids.

This study was conducted at 12 centers in Japan and performed in accordance with the principles of the Declaration of Helsinki [12] and the Japan Good Clinical Practice. The study was registered with the Japan Registry of Clinical Trials (https://jrct.mhlw.go.jp) as jRCT2071220040 and was approved by the Institutional Review Board of Nagasaki University Hospital, Hokkaido University Hospital, Hamamatsu University School of Medicine, University of Tsukuba Hospital, National Hospital Organization Kyushu Medical Center, Osaka Metropolitan University Hospital, Fukushima Medical University Hospital, Okayama Saiseikai Outpatient Center Hospital, Keio University Hospital, Nagoya University Hospital, Yokohama City University Hospital, and Hiroshima University Hospital. Participants were recruited from 22 July 2022 to 1 February 2024. They were randomly assigned in a 1:1:1 ratio to receive 5-ALA-HCl 100 mg/day + SFC 78.4 mg/day (low-dose), 5-ALA-HCl 300 mg/day + SFC 235.2 mg/day (high-dose), and placebo. Case registration and patient allocation were conducted at the Data Center of the Department of Medical Innovation, Osaka University Graduate School of Medicine, using a centralized registration system via the Case Registration Web System (Datatrak Enterprise Cloud). Allocation was performed randomly by the system's program (method: simple randomization), and allocation results were displayed using numbers that could not identify the allocation groups. Case registration and allocation were carried out in accordance with separate procedures specified on the website. No adjustment factors were set for allocation. The basis for determining the dosage of this drug in this clinical trial is as follows: In clinical trials targeting type 2 diabetes and cisplatin-induced nephropathy, the efficacy of 5-ALA-HCl at a dosage of 300 mg/day (150 mg twice daily) has been confirmed (trial numbers: NPJ005-DM2-0522 and SPP3C301). The mechanism of action of this drug is believed to contribute to efficacy through anti-inflammatory effects in type 2 diabetes via activation of the electron transport chain and induction of HO-1 expression, and in cisplatin nephropathy via induction of HO-1 expression [8,9,13], and since both mechanisms are considered to be similar to those of AOSD, it was expected that 300 mg/day would demonstrate efficacy for AOSD as well. Furthermore, since this clinical trial is a dose-finding study, a 100 mg/day dose group was established, and 100 mg/day and 300 mg/day were set. Regarding the administration period, since this agent reaches a steady state within one day, the administration period was set at eight weeks. The target enrollment was 30 participants (10 per group); however, the study was discontinued early after enrolling four participants. All participants who completed the double-blind phase were transferred to the 5-ALA-HCl arm and enrolled in an extension study (jRCT2071220086). Owing to early termination, this study presents pilot data from four participants. The small sample size limits generalizability, and the results should be interpreted as preliminary evidence that requires validation in larger, adequately powered studies.

Patients

Participants who met all of the following criteria were recruited: (1) patients who were 16 years of age or older at the time consent was obtained, any gender was acceptable; (2) patients who confirmed diagnosis of AOSD as per Yamaguchi criteria [14] with an onset of disease 16 years of age or older; (3) patients with a history of refractory to treatment with corticosteroids at a prednisolone equivalent of 0.4 mg/kg/day or more for at least two weeks; (4) patients with all of the following a-c disease activity during the screening period despite taking prednisolone equivalent of 10 mg/day or more at a fixed dose for at least one week prior to obtaining consent; a) Patients with fever> 38°C for even one day due to AOSD; b) Patients with an active joint count (tender (68 joints) or swollen (66 joints)) of two or more; c) Patients with ESR (Westergren method)> 20 mm/h or CRP> 1.0 mg/dL. (5) patients whose free informed consent could be obtained in writing and who are able to comply with the requirements of the study protocol; for subjects under 18 years of age, written consent must be obtained from a surrogate and, in principle, from the patient him/herself; (6) females of childbearing potential or males who are sexually active with females of childbearing potential who agree to use an effective contraceptive method (e.g., condom) for the duration of the study and until the day after the last dose of study drug.

The exclusion criteria were as follows: (1) patients classified as severe according to the AOSD severity classification of the Research Team for Autoimmune Diseases, the Research Program for Intractable Disease of the Ministry of Health, Labor and Welfare, Japan [15]; (2) patients who had previously taken 5-ALA/SFC; (3) patients who could not use appropriate contraception during the drug administration period of the study; (4) female patients during pregnancy or lactation; and (5) patients judged inappropriate for any other reason by the clinical investigator or clinical trial physician.

Interventions

Participants were randomized into three groups: 5-ALA-HCl 100 mg/day + SFC 78.4 mg/day (low-dose), 5-ALA-HCl 300 mg/day + SFC 235.2 mg/day (high-dose), and placebo. The study drug (5-ALA-HCl/SFC) was provided by KIYAN PHARMA Co., Ltd. It was administered orally twice daily for eight weeks. Following the administration of 5-ALA-HCl/SFC, participants who achieved adapted ACR 30 at the Week 4 and Week 6 follow-up visits were eligible for tapering of corticosteroids at the discretion of the physician. The corticosteroid tapering regimen was carried out in accordance with a pre-determined protocol. If an increase or re-increase in oral corticosteroid dosage was considered necessary, the study drug was discontinued.

During the study period, the initiation of the following treatments was prohibited: administration of immunosuppressants, biologics, or Janus kinase inhibitors; intra-articular corticosteroid injections at joints; and suppositories. An appropriate washout period was provided if these drugs had been administered in advance. The washout periods for each drug were as follows: Canakinumab, infliximab, and infliximab biosimilar were 12 weeks; golimumab, adalimumab, adalimumab biosimilar, certolizumab pegol, and abatacept were eight weeks; tocilizumab, sarilumab, and cyclophosphamide were four weeks. Etanercept and its biosimilar had a washout period of two weeks, as did Janus kinases (JAK) inhibitors. Immunosuppressive drugs such as cyclosporine, tacrolimus, azathioprine, and mycophenolate mofetil also had a washout period of two weeks.

Participants were discontinued prematurely if they met any of the following criteria: if the patient asked to leave the trial; if it was determined after registration that a subject was ineligible for the trial; if a physician determined that continuing the trial was difficult due to worsening of complications; if a physician determined that continuing the trial was difficult due to adverse events; if pregnancy was confirmed in a subject; if the entire clinical trial was discontinued; if any other serious violations of the clinical trial protocol were identified; if the principal investigator or the sub-investigator determined that termination was necessary.

Study endpoints

The primary endpoint was the achievement of adapted ACR 30 [3,16] at week 4. The adapted ACR 30 is a composite measure of disease activity. It was defined as meeting the following criteria: improvement of 30% or more in three or more of the following five items: 1) to 5) from baseline, with no fever exceeding 38°C in the past week, and no worsening of more than 30% in any of the following five items: 1) Physician-assessed global assessment of disease activity (VAS); 2) Patient-assessed disease activity (VAS); 3) Functional status assessed using the Health Assessment Questionnaire Disability Index (HAQ-DI); 4) Number of active joints (swollen joints and tender joints); 5) Laboratory values of ESR (mm/h) or CRP (mg/dL)

The secondary endpoints included the following: Adapted ACR 30 at weeks 2, 6, and 8; adapted ACR 50/ 70/ 90/ 100 at weeks 2, 4, 6, and 8; corticosteroid dose reduction at week 8; and changes in systemic feature scores (SFS), serum ferritin levels, and EuroQol 5-dimensions 5-levels (EQ-5D-5L) at weeks 4 and 8. Adverse events (AEs) were also collected as safety data. For adapted ACR 50/ 70/ 90/ 100, it is defined as meeting the following criteria: three or more of the five items listed in 1) to 5) above have improved by 50%, 70%, 90%, or 100%, there has been no fever exceeding 38°C in the past week, and no more than one item has worsened by more than 30% of the five items listed in 1) to 5) above. The SFS scoring system consists of five clinical and five laboratory assessments and was designed to evaluate systemic disease features [17]. It has also been used as a secondary endpoint in a phase III trial investigating the efficacy of tocilizumab in Japanese patients with AOSD [18].

Statistical analysis

We planned to study 30 participants. Due to the limited number of patients, conducting a large-scale verification trial was considered extremely difficult. Therefore, no statistical sample size design was performed for this trial, and the target number of cases was set at 30 (10 per group) based on feasibility considerations. This calculation was based on the assumption that approximately 550 patients with AOSD would receive outpatient care at the 11 major facilities and related facilities anticipated to participate in the trial. According to the national AOSD survey, 11.6% of patients were deemed by their physicians to have residual activity at the final examination [1]. Additionally, at the time of trial planning, around 20% of patients with AOSD at Nagasaki University Hospital were deemed to have residual activity by their physicians. Therefore, it was estimated that there would be approximately 80 patients (around 15% of the total) among the 550 patients with AOSD attending the 11 facilities (including affiliated facilities). In other clinical trials (UMIN000028010: randomized, double-blind, parallel-group comparison trial of tocilizumab for colchicine-resistant familial Mediterranean fever), approximately 40% of patients who underwent eligibility screening were enrolled. Based on this experience, it was assumed that 30 patients would be enrolled in the investigator-initiated clinical trial. But as this study was terminated early, the data for four participants were presented as the pilot data. Owing to early termination and small sample size, formal statistical analysis was not conducted. Descriptive summaries were provided for all the participants. Categorical variables are described as frequencies and proportions of the number of participants analyzed, and continuous variables are presented as medians with minimum and maximum values. Data analyses were performed using SAS version 9.4 (SAS Institute, Inc., Cary, North Carolina).

## Results

Patient flow and characteristics

**Figure 1:**
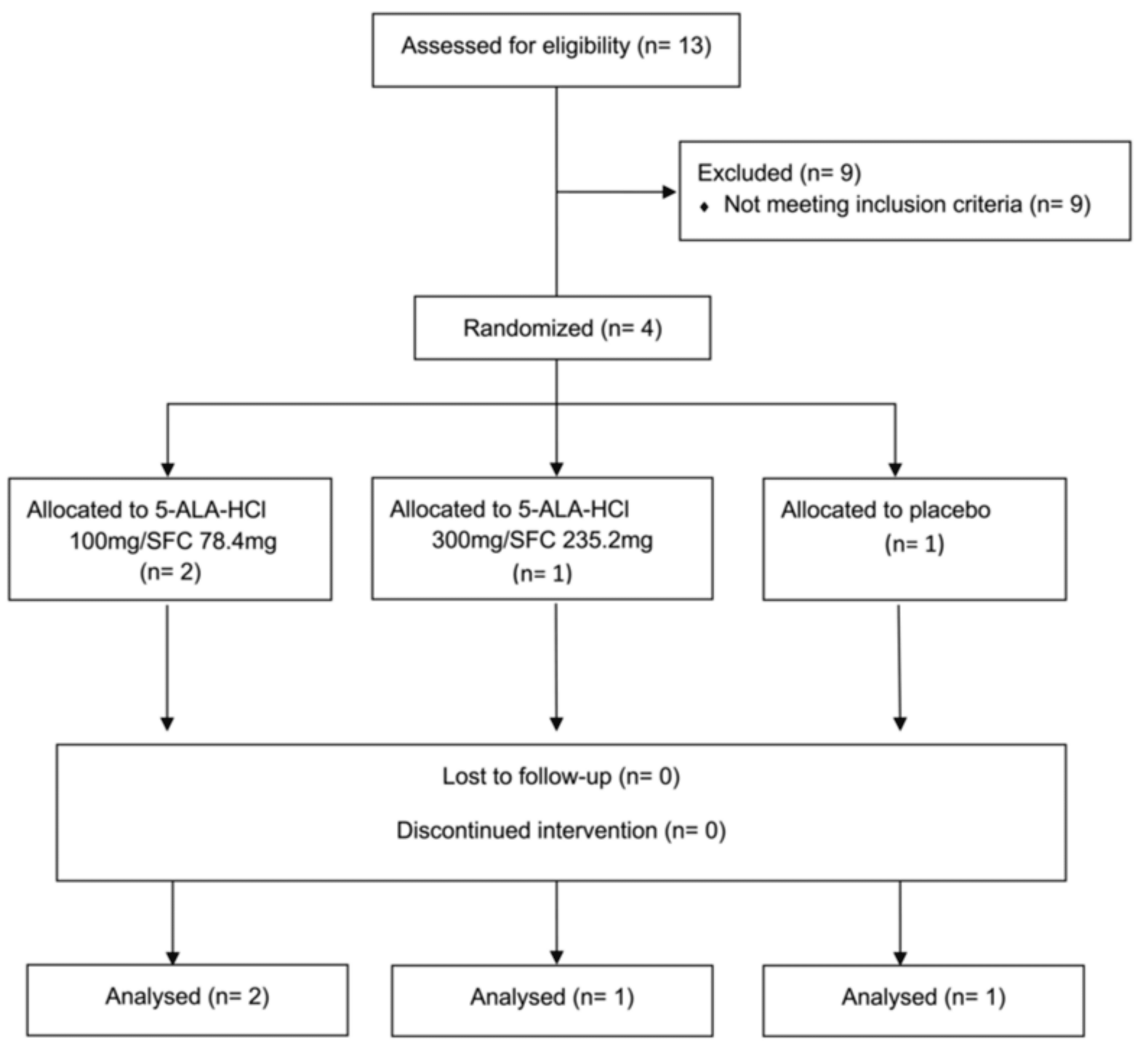
Patient flowchart SFC: sodium ferrous citrate; 5-ALA: 5-aminolevulinic acid

The trial was conducted from May 16, 2022, to April 30, 2024. Due to the impact of the COVID-19 pandemic, the recruitment of clinical trial participants did not proceed as planned. Owing to delays in participant enrollment and funding issues, the trial investigator decided to terminate the trial early. In this clinical trial, four participants were randomized from 13 participants who provided informed consent. Nine cases were excluded from the screening because they failed to meet all three of the following activity criteria during the screening period: a. fever exceeding 38°C; b. arthritis in two or more joints; and c. ESR of 20 mm/h or CRP >1.0 mg/dL. Figure 1 shows a flowchart of the study. Two participants were assigned to the 5-ALA-HCl 100 mg/day + SFC 78.4 mg/day (low-dose) group, one participant to the 5-ALA-HCl 300 mg/day + SFC 235.2 mg/day (high-dose) group, and one participant was assigned to the placebo group. All four randomized subjects completed the trial without discontinuing the study drugs during the trial period. Table 1 presents the baseline characteristics. The median age of the participants was 59.5 years old. Two of the four randomized participants were female. The median disease duration was 28.1 months. Of the four subjects, three had a history of treatment with drugs other than prednisolone, and one (in the placebo group) had no history of treatment with drugs other than prednisolone. The median dose of concomitant prednisolone was 19 mg/day. The median CRP, ESR, and ferritin levels were 3.23 mg/dl, 45 mm/h, and 2615 ng/mL, respectively. The median systemic feature score was 3.5, and the median EQ-5D-5L score was 0.73.

**Table 1:** Baseline characteristics of patients with adult-onset Still's disease treated with study drug (n=4) CRP: C-reactive protein; EQ-5D-5L: EuroQol 5-dimensions 5-levels; ESR: erythrocyte sedimentation rate; SFC: sodium ferrous citrate; VAS: visual analog scale; 5-ALA: 5-aminolevulinic acid

Characteristics	Median (Min-Max) or n (%)	Case 1	Case 2	Case 3	Case 4
Study drug		5-ALA-HCl 300 mg/day + SFC 235.2 mg/day	5-ALA-HCl 100 mg/day + SFC 78.4 mg/day	5-ALA-HCl 100 mg/day + SFC 78.4 mg/day	Placebo
Age, years	59.5 (19-77)	77	65	54	19
Sex, female, n (%)	2 (50%)	Female	Male	Male	Female
Body weight, kg	63.4 (44.4-88.0)	44.4	61.8	65	88
Disease duration, months	28.1 (6.2-145.2)	48.4	145.2	7.8	6.2
Previous treatment other than prednisolone, n (%)	3 (75%)	Yes	Yes	Yes	No
Methylprednisolone plus, n (%)	2 (50%)	Yes	Yes		
Tocilizumab, n (%)	1 (25%)		Yes		
Methotrexate, n (%)	1 (25%)		Yes		
Cyclosporine A, n (%)	2 (50%)		Yes	Yes	
Dose of concomitant prednisolone, mg	19 (10-30)	10	30	13	25
CRP, mg/dL	3.23 (2.19-7.4)	7.4	2.76	3.69	2.19
ESR, mm/h	45 (34-101)	101	41	34	49
Serum ferritin, ng/mL	2615 (278-4610)	4610	3360	278	1870
Systemic feature score	3.5 (3-5)	5	3	3	4
EQ-5D-5L (score)	0.73 (0.04-0.89)	0.04	0.78	0.68	0.89
EQ-5D-5L (VAS)	62.5 (20-75)	20	50	75	75

Primary endpoint

In this small pilot data (n = 4), all randomized participants achieved the adapted ACR 30 criterion at week 4 after administration of the study drug (high-dose group: n = 1; low-dose group: n = 2; placebo group: n = 1). Results are presented descriptively and are not intended to support statistical conclusions.

Secondary endpoints

Table 2 lists the secondary endpoints, CRP, and ESR levels for each case.

**Table 2:** Primary and secondary endpoints, CRP and ESR levels ACR: American College of Rheumatology; CRP: C-reactive protein; EQ-5D-5L: EuroQol 5-dimensions 5-levels; ESR: erythrocyte sedimentation rate; SFC: sodium ferrous citrate; VAS: visual analog scale; 5-ALA: 5-aminolevulinic acid

Endpoints		Case 1	Case 2	Case 3	Case 4
Study drug		5-ALA-HCl 300 mg/day + SFC 235.2 mg/day	5-ALA-HCl 100 mg/day + SFC 78.4 mg/day	5-ALA-HCl 100 mg/day + SFC 78.4 mg/day	Placebo
Achieved adapted ACR values	Baseline	-	-	-	-
	Week 2	Not achieved	70	Not achieved	70
	Week 4	70	70	50	70
	Week 6	90	70	Not achieved	50
	Week 8	90	70	Not achieved	70
Systemic feature score	Baseline	5	3	3	4
	Week 4	4	3	4	2
	Week 8	2	2	4	2
Serum ferritin (ng/mL)	Baseline	4610	3360	278	1870
	Week 4	5250	441	254	200
	Week 8	431	386	431	110
EQ-5D-5L scores	Baseline	0.04	0.78	0.68	0.89
	Week 4	0.89	0.94	0.89	0.76
	Week 8	0.94	0.94	0.76	0.76
EQ-5D-5L (VAS)	Baseline	20	50	75	75
	Week 4	95	90	90	86
	Week 8	99	90	50	75
CRP (mg/dL)	Baseline	7.4	2.76	3.69	2.19
	Week 2	7.15	0.39	6.25	0.07
	Week 4	3.45	0.39	3.88	0.02
	Week 6	1.05	0.75	8.18	0.06
	Week 8	1.04	0.31	8.92	0.07
ESR (mm/h)	Baseline	101	41	34	49
	Week 2	107	33	52	21
	Week 4	79	42	31	6
	Week 6	62	44	59	11
	Week 8	55	47	77	9

Adapted ACR 30 at weeks 2, 6, 8

The participant in the high-dose group (Case 1) did not achieve the adapted ACR 30 at week 2 but did so at weeks 6 and 8. One participant in the low-dose group (Case 2) and one in the placebo group (Case 4) achieved adapted ACR 30 at all time points. The other participant in the low-dose group (Case 3) did not achieve adapted ACR 30 at any time point.

Adapted ACR 50/ 70/ 90/ 100 at weeks 2, 4, 6, and 8

One participant in the high-dose group (Case 1) did not achieve adapted ACR 50 at week 2, but achieved adapted ACR 70 at week 4, adapted ACR 90 at week 6, and maintained adapted ACR 90 at week 8. One participant in the low-dose group (Case 2) achieved adapted ACR 70 at week 2 and maintained adapted ACR 70 at all subsequent time points up to week 8, but did not achieve adapted ACR 90 or higher. One participant in the low-dose group (Case 3) did not achieve adapted ACR 50 at week 2, achieved adapted ACR 50 at week 4, but did not achieve adapted ACR 50 at weeks 6 or 8. One participant in the placebo group (Case 4) achieved adapted ACR 70 at weeks 2 and 4 but did not meet adapted ACR 70 at week 6, achieving adapted ACR 50, and then achieved adapted ACR 70 again at week 8. Adapted ACR 90 or higher was not achieved at any time point. None of the participants achieved the adapted ACR 100.

Corticosteroids dose reduction

Among the four subjects, a dose reduction in glucocorticoids was observed in two subjects at week 8 compared to that at baseline. One participant in the low-dose group (Case 2) and one participant in the placebo group (Case 4) (30 mg to 17.5 mg/day and 25 mg to 15 mg/day, respectively). The other participant in the low-dose group (Case 3) and one participant in the high-dose group (Case 1) had the same dosage of corticosteroids at week 8 as at baseline (13 mg/day and 10 mg/day, respectively).

Changes in systemic feature scores (SFS), serum ferritin, and EQ-5D-5L

In one participant in the high-dose group (Case 1), the change in SFS was -1 and -3 at weeks 4 and 8, respectively (baseline/ week 4/ week 8: 5/ 4/ 2). In the low-dose group, one participant's (Case 2) SFS changed by 0 and -1, respectively (baseline/ week 4/ week 8: 3/ 3/ 2). For another participant in the low-dose group (Case 3), the SFS change was 1 (baseline/ week 4/ week 8: 3/ 4/ 4). In one participant in the placebo group (Case 4), the SFS change was -2 (baseline/ week 4/ week 8: 4/ 2/ 2).

We also observed changes in the serum ferritin levels. In one participant in the high-dose group (Case 1), the change in serum ferritin levels at weeks 4 and 8 was 640 ng/mL and -4,179 ng/mL, respectively (baseline/ week 4/ week 8: 4,610/ 5,250/ 431 ng/mL). In the low-dose group, one participant (Case 2) experienced a change in serum ferritin levels of -2,919 ng/mL and -2,974 ng/mL at weeks 4 and 8, respectively [baseline/ week 4/ week 8: 3,360/ 441/ 386 (ng/mL)]. For the other participant in the low-dose group (Case 3), the change from baseline to weeks 4 and 8 was -24 ng/mL and 153 ng/mL, respectively [baseline/ week 4/ week 8: 278/ 254/ 431 (ng/mL)]. In the placebo group (Case 4), one participant showed a change of -1,670 ng/mL and -1,760 ng/mL at weeks 4 and 8, respectively [baseline/ week 4/ week 8: 1,870/ 200/ 110 (ng/mL)].

Regarding changes in EQ-5D-5L scores, in the high-dose group (Case 1), the change in EQ-5D-5L scores at weeks 4 and 8 was 0.85 and 0.90, respectively (baseline/ week 4/ week 8: 0.04/ 0.89/ 0.94). In the low-dose group, the change in scores was 0.16 for one participant (Case 2) at weeks 4 and 8 (baseline/ week 4/ week 8: 0.78/ 0.94/ 0.94). In the other participant in the low-dose group (Case 3), the change in scores at weeks 4 and 8 was 0.21 and 0.08, respectively (baseline/ week 4 / week 8: 0.68/ 0.89/ 0.76). In the placebo group (Case 4), the change in scores at weeks 4 and 8 was -0.13 (baseline/ week 4 / week 8: 0.89/ 0.76/ 0.76). In the high- and low-dose groups, numerical improvements (increased scores) were observed in the changes in EQ-5D-5L scores from baseline at weeks 4 and 8. However, no improvements were observed in the placebo group.

In the EQ-5D-5L VAS (where 100 is the best possible state and 0 is the worst possible state), the change in the EQ-5D-5L VAS at weeks 4 and 8 in the high-dose group (Case 1) was 75 and 79, respectively (with baseline/ week 4/ week 8 scores of 20, 95, and 99, respectively). In the low-dose group (Case2), the EQ-5D-5L VAS changed by 40 at both weeks 4 and 8 (baseline/ week 4/ week 8: 50/ 90/ 90). For another participant in the low-dose group (Case 3), the EQ-5D-5L VAS changed by 15 and -25 at weeks 4 and 8, respectively (baseline/ week 4/ week 8: 75/ 90/ 50). For one participant in the placebo group (Case 4), the EQ-5D-5L VAS changes at weeks 4 and 8 were 11 and 0, respectively (baseline/ week 4/ week 8: 75/ 86/ 75). Numerical improvements (increased EQ-5D-5L VAS scores) from baseline at weeks 4 and 8 were observed in one participant in the high-dose group (Case 1) and one in the low-dose group (Case 2).

Safety

No AEs were observed during the observation period of this clinical trial in the four subjects who received the study drug (one in the high-dose group, two in the low-dose group, and one in the placebo group). No AEs were observed in this small cohort; however, safety needs confirmation in larger populations.

## Discussion

This trial is the first prospective randomized controlled trial to evaluate the efficacy and safety of 5-ALA-HCl/SFC in the treatment of AOSD. However, as the study was terminated early, data from only four participants were presented as pilot data. While the primary endpoint (adapted ACR 30) was achieved in all cases, the high-dose group showed a trend toward efficacy in several secondary endpoints compared with the other groups. The results of the subject assigned to the high-dose group were higher than those of the other three subjects at weeks 6 and 8 for the achievement of adapted ACR 90, the change from baseline in systemic feature score, the change from baseline in serum ferritin levels, and the change from baseline in EQ-5D-5L. The effects of concomitant corticosteroids should also be considered. However, participants in the high-dose group actually received a lower dose of corticosteroids than those in the other groups. While this suggests the efficacy of 5-ALA-HCl 300 mg/SFC 235.2 mg (daily dose), the efficacy of this treatment in patients with AOSD refractory to corticosteroids could not be demonstrated in this study, as the comparison could not be evaluated with the originally planned number of patients.

Currently, no well-established treatment for AOSD exists. Alternative therapies are needed because of AEs associated with the long-term use of glucocorticoids, biologics, and immunosuppressants. Based on our preclinical studies [19], 5-ALA/SFC showed preventive and therapeutic effects in collagen-induced arthritis mice without significantly affecting adaptive immune cells, acting primarily on macrophages and dendritic cells. It reduced key chemokines and shifted immune cell phenotypes from inflammatory to regulatory. Furthermore, 5-ALA/SFC suppressed systemic inflammation in a mouse model of AOSD, a systemic disease, and its severe form, macrophage activation syndrome, using C57BL/6N mice repeatedly administered CpG phosphorothioate oligodeoxynucleotides [19]. In a clinical study (jRCTs071190042) of patients with AOSD with inadequate response to standard therapy, supplementation with 5-ALA-phosphate/SFC was well tolerated and allowed for glucocorticoid reduction [19,20]. These results suggest that 5-ALA-HCl/SFC may be effective in treating AOSD. We considered the potential of 5-ALA-HCl/SFC to replace existing biologics and immunosuppressants, although its use in combination with these agents may also be feasible depending on the clinical context.

In this study, the primary endpoint was achieved in all participants. However, this may have been influenced by the very limited number of enrolled cases. The reason why one participant in the placebo group achieved the primary endpoint may include spontaneous improvement in disease activity, effects of concomitant corticosteroid therapy, placebo effect, or variability in joint assessment and patient-reported outcomes. Given the small cohort and variability inherent in AOSD, such findings should be interpreted with caution. Because the evaluation was based on only four cases, a major limitation was the heterogeneity in the participants’ background factors, such as age, weight, disease duration, prior medications, comorbidities, and clinical test results. Furthermore, the uneven allocation across treatment arms (one participant in the high-dose group, two in the low-dose group, and one in the placebo group) made it difficult to interpret efficacy trends, particularly regarding potential dose-response relationships. Therefore, the results should be considered descriptive and exploratory, and no definitive conclusions could be drawn about the relative efficacy of different doses. Another limitation was our inability to adequately assess selection bias due to the high screening failure rate (69.2%) and the limited data collected for the excluded participants. While basic demographic information (age and sex) was available for the nine excluded participants, detailed clinical characteristics were not systematically collected because of the early termination of the study and constraints on consent. This prevented a comprehensive comparison between the excluded and enrolled participants, which limited our ability to evaluate whether the small randomized cohort (n=4) represented a systematically different population. Future studies should implement systematic screening data collection from the outset to enable proper bias assessment and improve the evaluation of the generalizability of the results. Additionally, one of the reasons for the high screening dropout rate regarding the inclusion criteria was that participants had to be taking 10 mg/day or more of prednisolone and meet all of the following conditions during the screening period: fever, arthritis, and elevated inflammatory markers. Therefore, we should consider revising the residual disease activity criteria to include two or more of these conditions.

As there are no established clinical evaluation indices for AOSD, it remains to be determined whether this endpoint is appropriate. However, regarding the evaluation of efficacy, the adapted ACR, and the CRP and ESR values, and SFS values showed similar trends in the same participants. Additionally, although HO-1 levels were not assessed in this study, their measurement should be considered in future studies. No AEs were observed in any of the subjects, and the treatment was well tolerated by all. Although the observation period was only eight weeks, the efficacy and safety of the treatment were evaluated over a longer period in a 16-week extension study following this clinical trial (jRCT2071220086).

## Conclusions

While the efficacy of 5-ALA-HCl 300 mg/SFC 235.2 mg was suggested, the results of this clinical trial did not permit any conclusions to be drawn concerning the efficacy, safety, and risk-benefit balance of 5-ALA-HCl/SFC in patients with AOSD. This question needs to be addressed definitively in future research, which would require a well-designed and adequately funded study.
